# Pregnancy-Related Immune Changes and Demyelinating Diseases of the Central Nervous System

**DOI:** 10.3389/fneur.2019.01070

**Published:** 2019-10-09

**Authors:** Ke Qiu, Qiang He, Xiqian Chen, Hui Liu, Shuwen Deng, Wei Lu

**Affiliations:** Department of Neurology, The Second Xiangya Hospital, Central South University, Changsha, China

**Keywords:** demyelinating diseases, pregnancy, multiple sclerosis, acute disseminated encephalomyelitis, neuromyelitis optica spectrum disorders

## Abstract

Demyelinating diseases of the central nervous system comprise a heterogeneous group of autoimmune disorders characterized by myelin loss with relative sparing of axons occurring on a background of inflammation. Some of the most common demyelinating diseases are multiple sclerosis, acute disseminated encephalomyelitis, and neuromyelitis optica spectrum disorders. Besides showing clinical, radiological, and histopathological features that complicate their diagnosis, demyelinating diseases often involve different immunological processes that produce distinct inflammatory patterns. Evidence of demyelination diseases derives mostly from animal studies of experimental autoimmune encephalomyelitis (EAE), a model that relies on direct antibody–antigen interactions induced by encephalitogenic T cells. Pregnancy is characterized by non-self-recognition, immunomodulatory changes and an altered Th1/Th2 balance, generally considered a Th2-type immunological state that protects the mother from infections. During pregnancy, the immune response of patients with autoimmune disease complicated with pregnancy is different. Immune tolerance in pregnancy may affect the course of some diseases, which may reach remission or be exacerbated. In this review, we summarize current knowledge on the immune status during pregnancy and discuss the relationship between pregnancy-related immune changes and demyelinating diseases of the central nervous system.

## Introduction

Demyelinating diseases of the central nervous system (CNS) comprise a group of autoimmune disorders, including multiple sclerosis (MS), acute disseminated encephalomyelitis (ADEM), neuromyelitis optica spectrum disorders (NMOSD), and other secondary CNS inflammatory demyelinating diseases (1, 2). The first description of MS by Charcot dates back to 1868; later, in 1894, Devic coined the term acute optic neuromyelitis (3).The emergence of autoantibody detection technology has enabled a better understanding of demyelinating diseases of the CNS. In 2004, aquaporin-4 was identified as the target of NMO, and a diagnostic test for NMO based on the detection of anti-aquaporin-4 (AQP4) autoantibodies (NMO-IgG) was developed (4). The spectrum of clinical features in NMO has broadened in the past years (4, 5). Anti-myelin oligodendrocyte glycoprotein (MOG) antibodies are found in patients with ADEM, NMOSD, and MS-like disease (6, 7), and one study has reported anti-MOG antibodies in approximately 20% of patients with ADEM (8). Recent studies indicated that MOG antibodies are present in some patients with AQP4 negative NMOSD (9) and in only very few patients with demyelinating syndromes associated with anti-*N*-methyl-d-aspartate (NMDA) receptor antibodies (10). The diversity in clinical features may depend on the different underlying immunological mechanisms. Overall, demyelination and neurodegeneration in demyelinating diseases of the CNS are triggered through different mechanisms, and show different progression of disease characteristics. These are summarized in Table 1. Further research is needed to explore the link between demyelinating diseases.

**Table 1:** Mechanisms of demyelinating diseases of the central nervous system.

**Forms**	**Disease mechanism**
Relapsing MS	Relapsing MS is driven by immune cells that migrate to CNS. Currently there are a variety of treatments by intervention of these pathways reduce the recurrence of MS: reducing the number/function of effector cells, increasing the number/ function of regulating cells, preventing cells from being transported to the CNS
Progressive MS	The immune response in the central nervous system is dominant in the progressive stage, as well as immune independent is included. A micro-environment is created within the CNS favoring homing and retention of inflammatory cells (B cells, pro-inflammatory cytokines), causing disease-modifying therapies to ineffective
ADEM	Two hypotheses for the pathogenic of ADEM: The molecular mimicry and The postinfectious etiology hypothesis. The changes of immune system during pregnancy will inevitably lead to changes in cytokine levels. Plasma exchange may remove harmful circulating antibodies that can alleviate clinical symptoms
NMOSD	The peripheral circulation contains auto-antibodies against aquaporin-4 (AQP4-IgG). AQP4-IgG enter to the CNS across the BBB cause a series of pathological changes. Maternal placenta can expression of AQP 4, leading to AQP 4 antibody-mediated placental attack, may also be a causative factor

*MS, multiple sclerosis; ADEM, Acute disseminated encephalomyelitis; NMOSD, Neuromyelitis optica spectrum disorders; AQP4, anti-aquaporin-4; CNS, central nervous system*.

Childbearing women are more predisposed to demyelinating diseases of the CNS (11). However, the immunological mechanisms of demyelinating episodes during pregnancy are not entirely clear. In this review, we summarize current knowledge on the immune status during pregnancy and discuss the relationship between pregnancy-related immune changes and demyelinating diseases of the CNS.

## Pregnancy and the Immune System

Pregnancy represents an exceptional challenge to the human immunological system—not because of the wide range of immune suppression, but rather due to the unique immune-tolerant condition. The maternal-fetal interface comprises the fetally derived placenta and the maternally derived decidua. Successful pregnancy involves complex interaction between decidual immune cells and trophoblast cells, which allow the semi-allogeneic fetus to evolve inside the mother's body without being attacked by the maternal immune system (12, 13). Uterine natural killer cells, T cells, immature dendritic cells, and macrophages help regulate the uterine environment to maintain a successful pregnancy. Once the maternal-fetal immune is disturbed, it can lead to pregnancy-related diseases with adverse pregnancy outcomes for both the mother and her fetus (14).

### Normal Changes of the Maternal Immune System During Pregnancy

The maternal-fetal interface is the interface between the uterine mucosa and the extraembryonic tissue (15). The fetal cells are in direct contact with the maternal immune system through the fetus' extravillous trophoblasts (EVTs). EVTs evade maternal immune surveillance while inducing immune tolerance by expressing a unique set of major histocompatibility complex (MHC) molecules (16). This is one of the specific mechanisms which exist to balance the maternal immunological system, so that the mother does not reject her fetus. The MHC is located on chromosome 6 and encodes HLA molecules, including HLAI, II and III. HLA class I includes classical antigens (HLA-A, HLA-B and HLA-C) and non-classical HLA class Ib antigens, including HLA-E, -F, and -G) (17). During pregnancy EVTs can express HLA-C, HLA-E, and HLA-G (18), but do not express HLA class I and II molecules or HLA-A and HLA-B (17). HLA-G is a non-classical MHC class I gene with a low degree of polymorphism, which seems to play a key role in the immunological mechanisms that control maternal–fetal tolerance during pregnancy (19). HLA-G is highly expressed in trophoblasts (20), where it induces an immune chemotactic response. HLA-G can inhibit the cytotoxic activity of natural killer (NK) cells and downregulate Th1 type cytokine production (21).

NK cells are the most abundant immune cell population in the decidua. In mice, they are recruited to the implantation site during decidualization. During pregnancy, extensive uterine remodeling, cell proliferation, and cell invasion occur (22). The uterine NK (uNK) cells can prevent a fully activated inflammatory response, limit trophoblast invasion, and maintain decidual and spiral artery integrity.

Accumulating evidence has shown that lymphocytes at the maternal–fetal interface are activated and release various cytokines (23). Since the 1980s, the immune status of normal pregnancy has been conceptualized as a transition from a T helper 1 (Th1) to a T helper type 2 (Th2) immune environment (24, 25); that is to say, a functional change in conventional T cells occurs to maintain fetal tolerance. Th1 cells participate in cellular immunity by secreting TNF-α, TNF-β, IFN-γ, and IL-2. Th2 cells participate in humoral immunity by secreting IL-4, IL-5, IL-6, IL-10, IL-13, and transforming growth factor (TGF)-β (26–28). Th1 cells have been shown to be harmful to pregnancy. Administration of TNF-α, IFN-γ or IL-2 to normal pregnant mice results in miscarriage (29). In the murine model for spontaneous abortion, it has demonstrated that maternal strain cells respond to the stimulation provided by placental antigens through the production of IL-2, TNF-a, and IFN-γ *in vitro* (30). In normal pregnancy, the levels of serum Th2 cytokines IL-6 and IL-10 were found to be significantly higher than in patients with recurrent spontaneous abortion, while levels of serum Th1 cytokine IFN-γ is significantly elevated in recurrent spontaneous abortion (31). Interleukin-4 and IL-10 secreted by Th2 cells have been shown to support pregnancy, whereas tumor necrosis factor (TNF)-α, interferon (INF)-γ, and IL-2 secreted by Th1 cells are detrimental to fetal development in mice and humans (23, 24, 32). More and more evidence has shown that successful pregnancy is a Th2-type immunological state (23, 32, 33) that supports the implantation and survival of the fetus. A summary of normal changes in immune molecules in normal pregnancy is provided in Table 2.

**Table 2:** Normal changes in immune molecules in normal pregnancy.

**Immune pathway**	**Normal pregnancy**
Th1, Th2 balance	Th2 shift
IL-4, IL-6, and IL-10	Elevated
TNF-α, INF-γ, and IL-2	Reduced
Th17	Reduced
HLA-G	Elevated
NK cells	Elevated
IFN	Reduced IFN-γ
T-reg cells	Elevated

*Th, T-helper; HLA-G, major histocompatibility complex; NK, natural killer; IFN, interferons; TNF, tumor necrosis factor; T-reg, T regulatory*.

### Abnormal Changes of the Maternal Immune Function During Pregnancy

The relationship between pregnancy and autoimmune diseases has puzzled immunologists. Symptoms of autoimmune diseases may ameliorate, deteriorate, or show no changes at all when a woman is pregnant, depending on her unique disease. Pregnancy improves autoimmune diseases related to cell-mediated immunity, such as rheumatoid arthritis (RA). In one study, the symptoms of RA improved during pregnancy in 48–75% of patients and worsened after delivery in 41% of patients (34). The immunologic factors involved are not clear. A possible explanation for the improvement is that the normal placental biology can drive maternal tolerance to fetal antigens. However, pregnancy may worsen or have no effect on autoimmune diseases related to antibody-mediated immunity, such as systemic lupus erythematosus (SLE) (35, 36). Some degree of disease activity is thought to be present in 40–50% of SLE patients, with common manifestations such as lupus nephritis, arthritis, cutaneous disease, and hematologic disease (37). Moreover, the mother's autoimmune response could target the fetus and cause neonatal lupus syndrome when the auto-antibodies cross the placenta (35). While the clinical course of myasthenia gravis (MG) during pregnancy is variable; equal numbers of patients remain the same, improve, or worsen. After delivery, one-third of patients with MG experience exacerbations during the first 3 weeks (38). The potential roles for maternal immunologic factors are key to understanding the effect of pregnancy on the disease courses and the fetus.

## MS and Pregnancy

### MS

MS is an autoimmune neuroinflammatory disorder of the CNS (39, 40). It is characterized by a relapsing- remitting or chronic progressive disease course (39), in which both adaptive and innate immune systems participate in demyelination and neurodegeneration (41–43). The most common form is relapsing-remitting multiple sclerosis (RRMS), which affects approximately 85% of patients (44). For unknown reasons, MS is predominant among women, with onset at childbearing age (45). According to epidemiological data, about three-quarters of patients with MS are women.

The exact causes of MS are largely unknown (27, 46, 47). However, its development may be related to environmental exposure and genetic susceptibility, triggered by genetic polymorphisms (11, 39). There have been reports of a relationship between MS and specific HLA alleles, strongly driven by variants in HLA-DRB and HLA-A (48). It was found that HLA-DRB1^*^1501 is the most consistently identified genetic marker of MS susceptibility (49), followed by the genes encoding the α-chains of the IL-2 and IL-7 receptors (44). However, genes are not the only disease determinant; the combined action of the environment and genes results in the destruction of peripheral immune tolerance against CNS antigens involved in MS pathogenesis (48).

### T Cells and Molecular Theory Mechanism

At the cellular level, MS is caused by the activation of peripheral autoreactive effector CD4 T cells, that migrate into the CNS and initiate the disease process (50, 51). Together with T cells, antibodies may also contribute to MS (52). In patients with MS, the CNS is filled with inflammatory cuffs formed by infiltration of lymphocytes and macrophages, which provide the necessary cytokines for the immune response. These cytokines contribute to a complex network system by interacting with each other.

Studies on MS have been focusing on the activation of autoreactive T cells accompanied by the functional disequilibrium of Th1 and Th2 cells (47, 53). MS is regarded as a Th1-mediated disease (42, 54). Th1 cells release mediators that cause an autoreactive inflammatory attack contributing to myelin degeneration, which is believed to be involved in the pathogenesis of MS (55). A T-cell-mediated cross-activation response is generated against myelin proteins, such as myelin basic protein (MBP), myelin oligodendrocyte protein (MOG), and proteolipid protein (PLP) (56), through a mechanism of molecular mimicry. Then, myelin-reactive T cells could across the BBB. At the same time, a genetic defect or polymorphism may cause primary susceptibility of the oligodendrocytes to immune injury (39, 52). Subsequently, T-cell and B-cell infiltrates and axonal injury disseminate, causing both white and gray matter atrophy (57, 58). It is elusive as to exactly how these intrinsic CNS events occur. Possible hypotheses are partly based on emerging insights into CNS immune surveillance, such as inflammatory responses to CNS viral infection, or subsequent primary neurodegeneration similar to that involved in Alzheimer's or Parkinson's disease (44).

### Changes in the Blood–Brain Barrier (BBB) in MS

**Figure 1:**
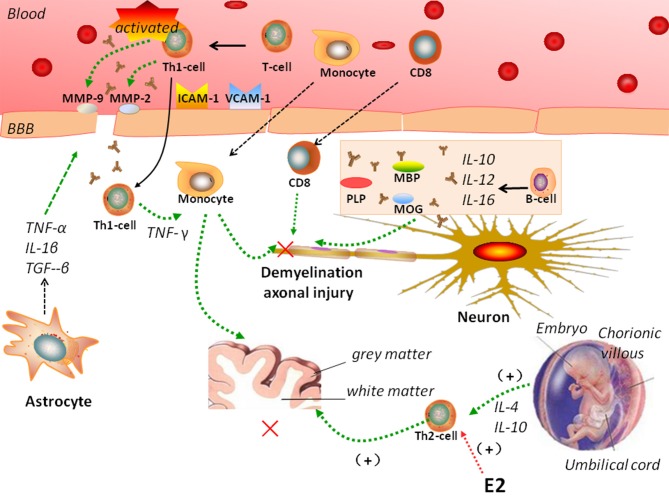
Complicated mechanisms of autoimmunity of multiple sclerosis (MS). In MS, peripheral auto-reactive T cells enter the central nervous system (CNS) via a disrupted blood-brain barrier (BBB). Vascular cell adhesion molecule (VCAM)-1, intercellular adhesion molecule (ICAM)-1, matrix metalloproteinase (MMP)-2, MMP-9, and astrocytes regulate BBB permeability. B cells, monocytes, and CD8 T cells participate in the pathogenesis of MS. MS is a Th1-mediated autoimmune disease, but pregnancy is characterized by a Th2-type immune state. Interleukin (IL)-4 and IL-10 secreted by Th-2 cells have been showed to support pregnancy. Estradiol and other sex hormones could influence the development of MS.

MS is characterized by a crosstalk between the adaptive and innate immune systems (Figure 1). In MS, peripheral auto-reactive T cells enter the CNS via a disrupted BBB and are responsible for inflammatory demyelination. Complement and TNF-α also participate in demyelination (47), and antibodies activate microglial cells and macrophages. The entry of peripheral autoreactive T cells into the CNS depends on their activation status, their ability to respond to cytokines and chemokine signals that induce their passage through the BBB, and the expression of adhesion molecules such as matrix metalloproteinase (MMP)-2, MMP-9, vascular cell adhesion molecule (VCAM)-1, and intercellular adhesion molecule (ICAM)-1. Investigators have identified Th17 cells as a new lymphocyte subset that drives inflammation by secreting IL-17, which can disrupt the BBB. Then, Th17 cells can penetrate the brain, where they induce neuronal damage (39).

During inflammation, astrocytes secrete TNF-α, IL-1β, and TGF-β onto endothelial cells to tight junctions and regulate BBB permeability. They also release MMPs, including MMP-2 and MMP-9, which further tighten the BBB (53). Astrocytes help prevent the migration of T cells across the glia limitans, impeding their way into the CNS (59, 60).

### The Interaction Between MS and Pregnancy

It is hard to reach a consensus regarding constitutive maternal immunity in patients with MS during pregnancy (61). The role of pregnancy on the long-term disease course and disability in MS is not yet clear, but it appears to be benign (62). During pregnancy, the differentiation of CD4^+^ T cells shifts from Th1 to Th2, and the production of Th2 cytokines increases (63), which inhibits the development of inflammatory Th1/Thl7 cells. The feto-placental unit also secretes cytokines that downregulate other cytokine-like substances mediating the mother's cellular immunity (28). These changes may explain the improvements of the clinical symptoms of MS during pregnancy.

A 1995 study revealed that there might be a link between pregnancy and a lower risk of onset of MS (64). However, some epidemiological studies have shown that pregnancy has no effect on the long-term outcome of MS (65, 66), whereas only one small study indicated a decreased risk of MS progression during pregnancy (64). The AusImmune Study revealed that the protective effect on MS risk is observed only in women. There may be a potential biological association with pregnancy-related changes in the mother (67). About 25 and 30% of women suffer relapses during the 40 weeks of pregnancy and the 3 months following delivery, respectively (68). Therefore, the safety of pregnancy in women with MS must be fully considered. Mueller designed a population-based cohort study for 198 women with MS and 1,584 women without MS. This study showed that women with MS were not more likely to have pregnancy or delivery complications, infants with low birth weight, preterm delivery or fetal malformations (69). An observational cohort study of 115 patients and 216 pregnancies (among 84 women) reported that the rate of pregnancy may be increasing in women with MS. However, this study also showed similar rates of spontaneous pregnancies per woman, time to pregnancy and spontaneous miscarriage rates as in the general population (70).

The literature on risk factors for MS during pregnancy has grown recently, with studies examining reduced sunlight exposure/vitamin D levels (71), cigarette smoking (72, 73), prenatal and perinatal factors (72), and breastfeeding (74–77). It is difficult to establish comprehensive multiple risk factors. We review the literature on possible risk factors for MS during pregnancy in Table 3.

**Table 3:** Summary of the correlation between risk factors and MS during pregnancy.

**Study, location**	**Design**	**Participants**	**Factors**	**Results**
Gardener et al. (72), United States	Retrospective	participants in the Nurses' Health Studies-2 prospective cohorts	Maternal nor paternal smoking; diabetes; maternal weight gain	Neither maternal nor paternal smoking was associated with a significantly risk of MS (RR for mothers: 0.97; 0.77–1.21 and forfathers 1.50; 0.99–2.28); diabetes during pregnancy (RR: 10; 2.5–42); maternal pre-pregnancy overweight /obesity (RR: 1.7; 1.0–2.7)
Montgomery et al. (73), Swedish	Case-control	143 cases/1,730 controls	Maternal smoking	No association between maternal smoking during pregnancy and risk of MS in the offspring (OR: 0.96, 0.65–1.44)
Langer-Gould et al. (71), NA	Case-control	5,296 cases/26,478 controls; 28 women with MS during pregnancy and at 2, 4, and 6 months post-delivery	Breastfeeding on 25(OH)D levels	Serum (25(OH)D) levels rose in women who breastfed non-exclusively compared to breastfed exclusively (*p* = 0.02); reduced 25(OH)D levels were not associated with an increased risk of postpartum MS relapse
Hellwig et al. (74), NA	prospective	201 patients with MS	The effects of breastfeeding on MS relapse rates	A significant association with breastfeed exclusively for at least 2 months with a reduced risk for postpartum relapses
Pakpoor et al. (77), NA	NA	869 breastfed MS/689 non-breastfed MS	The effects of breastfeeding on MS relapse rates	Women with MS who breastfed at a significantly reduced risk of a post-partum relapse compared to non-breastfed (OR: 0.53, 0.34–0.82). The authors noted significant heterogeneity across studies (*p* = 0.002)
Finkelsztejn et al. (78), NA	meta-analysis	Data from 13 studies, including 1,221 pregnancies	The effects of pregnancy on MS relapse rates	A significant decrease in relapse rate was observed during pregnancy; increase in the 3–12 months post-delivery: 0.76 (95% CI 0.64–0.87); the year prior to pregnancy:0.44 (95% CI 0.39–0.48); during pregnancy: 0.26(95% CI 0.19–0.32)
Vukusic and Confavreux (28), 12 European countries	prospective	With 227 pregnant women with MS and a full-term delivery of a life infant	The 2-year post-partum follow-up and the factors predictive of relapse in the 3 months after delivery	A lower risk of relapse during the 3rd trimesterr of pregnancy (*p* < 0.001), and a higher risk in the first 3 months post-delivery (vs. the year before pregnancy). The ARR: pre-pregnancy 0.7 (95%CI: 0.6–0.8); third trimester: 0.2 (0.2–0.3); 3 months post-delivery: 1.2 (1.1–1.4)
Confavreux et al. (79), NA	the seminal multinational study	254 women with MS	The effects of pregnancy on MS relapse rates	The ARR dropped from 0.7 per women per year (in the pre-pregnancy period) to 0.2 (in the third trimester); the relapse rate increased again during the first 3 months postpartum, reaching 1.2 per woman per year

*MS, multiple sclerosis; IRR, incidence rate ratio; ARR, annualized relapse rates; CI, confidence interval; OR, odds ratio; NA, not applicable*.

Women experience MS twice as frequently as men; changes in sex hormones has a great impact on the severity of MS (8, 80). The fluctuation of disease activity has suggested that sex hormones modulate autoimmunity (81). During pregnancy, especially in the third trimester, disease activity is at its lowest level (27, 82, 83). Levels of estrogens (estradiol and estriol) and progesterone gradually increase, and reach their peak in the third trimester. After birth, levels of these hormones fall, with a higher risk of MS in the post-delivery period, making their temporal profile consistent with the MS relapse rate (84). This is explained by the fact that high levels of estrogens and progesterone offer protection against disease activity during pregnancy (45).

Confavreux et al. discovered that relapse rates in MS are decreased during late pregnancy, when circulating estrogen (estradiol and estriol) levels increase (48, 79). In studies on EAE, Morales et al. (85) used this experimental model, with high doses of estradiol (to induce serum pregnancy hormone levels) and a highly selective estrogen receptor (ER)-α ligand *in vivo*, propyl pyrazole triol, as the positive control, to show that the clinical severity of EAE decreases following administration of propyl pyrazole triol (62, 85, 86). This indicated that ER activation may have a role in MS.

Estrogens can induce cytokine changes consistent with a Th1 to Th2 shift when administered *in vitro* to human immune cells and *in vivo* to mice (55, 85). One study from Iran investigators used female C57BL/6 mice immunized with MOG35–55 to show that, in splenocytes and lymph nodes, E2 implantation resulted in the production of equivalent levels of cytokines, such as TNF-α, IL-6, IL-17, and IFN-γ (pro-inflammatory cytokines), to those of pregnant mice, but lower than those of wild-type and placebo-implanted mice. On the contrary, the production of IL-4, IL-10, and TGF-β (anti-inflammatory cytokines) by splenocytes was higher in E2-implanted mice than in the other groups. That observation was consistent with the theory of a Th1 to Th2 shift (87). However, another study has shown that estrogens play a role in neuroprotection. This effect was mediated by ERα signaling via ERα on astrocytes and decreased expression of chemokine (C-C motif) ligand (CCL)-12 and CCL7 by astrocytes in EAE, but not via ERβ signaling on astrocytes and neurons (86). However, in the peripheral immune system, the expression of ERα was dispensable for the therapeutic effect. There has been an increasing concentration on the CNS targets of estrogens.

Several studies have investigated the prevention and treatment of MS by estrogen administration. Large placebo-controlled clinical trials of estrogen treatment in women with MS are ongoing, including a multicenter placebo-controlled phase 2 trial on estriol treatment in women with RRMS. The primary outcome was that estriol might play a role in decreased relapses (88). Another trial is examining the effects of estradiol and progestin therapy in preventing postpartum relapses of MS (89).

There is a need for better understanding of the effects of hormones on the immune system and the CNS, in order to target treatment strategies effectively. The aim is to protect the pregnancy and prevent harmful effects during the postpartum period.

## ADEM and Pregnancy

### ADEM

ADEM is an immune-mediated inflammatory demyelinating disease (56, 90–92). Distinct from multiple sclerosis, ADEM is characterized by a monophasic course, affects mostly children (93), and is more prevalent among men (90, 94–96). There is general agreement on the seasonal onset of ADEM in winter and spring (94).

The pathogenesis of ADEM is similar to that of EAE, which is mediated by auto-reactive CNS-specific T cells (90, 92). The demyelination is due to a transient autoimmune response toward myelin or other self-antigens.

The following hypotheses for the pathogenic of ADEM have been put forward (92, 97): (1) The molecular mimicry hypothesis: Viral or bacterial epitopes are similar to myelin antigens, and the structural similarities can lead to T-cell activation, but not sufficiently so to induce tolerance. However, the activated auto-reactive T cells can enter the CNS during ordinary immune surveillance. When they encountered the homologous myelin protein, a specific autoimmune response against the presumed foreign antigen is triggered (92, 98): (2) The postinfectious etiology hypothesis: A direct infection damages the tissue and disrupts the BBB (99). BBB disruption results in dysfunction, and systemic leakage of CNS-confined autoantigens into circulation perpetuates breakdown of tolerance with a self-reactive and encephalitogenic T-cell response (98).

Th2-related chemokines are thought to be produced in ADEM (100), resulting in neutrophil activation. Ichiyama et al. (101) found that the cerebrospinal fluid (CSF) concentrations of IL-6 and soluble TNF receptor 1 (sTNFR1) are elevated in ADEM, suggesting that these pro-inflammatory cytokines play a role in the pathogenesis of ADEM. Pro-inflammatory cytokines can cause CNS inflammation (99). Increased numbers of IL-γ-producing CD3^+^ cells have been found during the acute stage of ADEM (102).

### The Interaction Between ADEM and Pregnancy

The post-infectious etiology hypothesis of ADEM has been associated with trivial infection and vaccines (90), it is general believed that it seems support to original infection not the direct autoimmune response. As it may be associated with the changes in the immune system, changes in the immune status during pregnancy may cause worsening of ADEM symptoms. To the best of our knowledge, only four cases of ADEM complicated with pregnancy have been reported. Shah et al. (103) reported the first case: a woman in the late third trimester of pregnancy who developed the symptoms of ADEM, whose neurological status continued to deteriorate on high-dose corticosteroids. After corticosteroids failed to improve her condition, the patient was treated with plasmapheresis, and the symptoms improved significantly for a period of time, showing that plasma exchange was important for improving the symptoms of ADEM. Gaudry et al. (104) illustrated another case of ADEM diagnosed at 6 weeks of gestation. The patient had severe neurological impairment and cognitive symptoms. She was given both high-dose methylprednisolone and plasma exchange, and her neurological and psychiatric symptoms improved. There was a deterioration in neurological status 2 years after cesarean section at 33 weeks. Kaur et al. (105) reported a pregnant patient who was first misdiagnosed with mental illness, for which she was prescribed pharmacotherapy with olanzapine and lorazepam, without any improvement of the symptoms. After being diagnosed with ADEM, she was given intravenous (IV) steroids, IV immunoglobulin (Ig), and plasma exchange, without clinical progress. Her neurological status began to improve only after the pregnancy was medically terminated. Macerollo et al. (106) reported the newest case of ADEM complicated with pregnancy. The first symptoms appeared in the first trimester of pregnancy and were associated with cytomegalovirus infection. Despite aggressive treatment, her neurological status continued to deteriorate. Subsequently, she had craniotomy due to cerebral edema; medical abortion and plasmapheresis were also performed. Eventually, the symptoms disappeared gradually, and the level of consciousness improved completely. A summary of the four cases, including clinical symptoms, response to treatment, and pregnancy outcome, is shown in Table 4.

**Table 4:** Main characteristics of three cases of acute disseminated encephalomyelitis (ADEM) during pregnancy.

**Year**	**Age, years**	**Gestational age, weeks**	**Clinical features**	**Response to treatment**	**Long-term outcome**
2000	31	32	Bifrontal pressure-like headache; right-sided hemiparesis; deteriorated vision	Non-responsive to high-dose intravenous corticosteroids; responsive to plasmapheresis	Visual field deficits continued to improve; 9 months after giving birth, at an interim follow-up visit, there were no new neurological symptoms
2006	27	17	Left-sided hemiparesis and ataxia at onset, followed by tetraplegia, generalized seizure, and coma 3 days later	Responsive to high-dose intravenous methylprednisolone	Cesarean section at 33 weeks; spastic paraparesis with left predominance at 20 weeks, with ambulation limited to 15 m (requiring a walking aid), sensitive ataxia, Broca's aphasia, and amnesia
2014	23	6	Strange behavior; cognitive impairment; depression	Pharmacotherapy with olanzapine and lorazepam; intravenous steroids; intravenous immunoglobulins; plasmapheresis	Pregnancy terminated medically 1 month after admission; improved speech and comprehension
2016	40	4	Severe occipital headache; vision disturbance; rapid worsening of consciousness; epileptic seizures	Responsive to plasmapheresis	Improved symptoms and complete restoration of consciousness; the patient became able to stand and walk a few steps with assistance

The common features of these cases are recurrent symptoms during pregnancy, and improvement of the clinical symptoms of ADEM following plasma exchange. Since ADEM is similar to EAE, plasma exchange may remove harmful circulating antibodies that elicit demyelination, thus alleviating clinical symptoms. In addition, the clinical symptoms of ADEM were relieved after delivery or termination of pregnancy. However, the effect of pregnancy or termination of pregnancy on ADEM remains unknown (106). Previous studies found that the pathogenesis of ADEM is related to changes in cytokine levels (101). We speculate changes in the immune system during pregnancy will inevitably lead to changes in cytokine levels. Increased production of some cytokines may induce or exacerbate ADEM. Another possible explanation is that a compromised immune system in pregnancy may increase the risk of infection and subsequent autoimmune response. Viruses, such as the measles virus, smallpox, and influenza, have been suggested as trigger factors of ADEM. Because of the lack of retrospective and prospective studies, the interaction between ADEM and pregnancy warrants further study.

## NMOSD and Pregnancy

### NMOSD

NMOSD a group of demyelinating disorders of the CNS (107) with a wider definition than that of NMO (108), which predominantly involves the optic nerve and spinal cord (107, 109, 110). In 2004, Lennon et al. (5) identified pathogenic autoantibodies against AQP4 (NMO-IgG) as a highly specific marker of NMOSD. This marker predicts relapses, which play a role in the pathogenesis of NMOSD (108, 109, 111). AQP4-IgG-seropositive patients have a high risk of relapse with recurrent optic neuritis or longitudinally transverse myelitis (111). The incidence of NMOSD among women is higher than among men, ranging from 0.52 to 4.4/100,000 in different studies (108).

NMOSD is a severely disabling inflammatory disease (112) that is generally accepted to occur via a humoral autoimmune mechanism. Cytokines and chemokines have been proven to play important roles in the pathogenesis of NMOSD. T cells and B cell are also correlated with the pathogenesis of NMOSD. Studies have shown that Th17 and Th2 cytokines are upregulated in the CSF and serum of patients with NMOSD (113). In recent studies, the serum levels of three cytokines (IL-25, IL-31, and IL-33), which had been newly discovered to be Th2-related, were also shown to be increased in patients with NMOSD (114).

One hypothesis for the pathogenesis of NMOSD involves the entry of AQP4-IgG into the CNS, where it impairs BBB integrity (115). AQP4-IgG participates in the inflammatory cascade, possibly promoting oligodendrocyte injury and demyelination. AQP4-IgG also induces the activation of the complement system, thus increasing BBB permeability and antibody-dependent cellular cytotoxicity (also involving complement-dependent cellular cytotoxicity mechanisms). This eventually leads to demyelination and neuronal loss (116). Alternative NMO pathogenesis mechanisms include excitotoxicity-induced injury.

The expression of AQP4 is downregulated in acute spinal cord lesions, confirming the role of NMO-IgG in the pathogenesis of NMOSD. The expression of glial fibrillary acidic protein (GFAP), a marker of astrocytes, parallels the loss of AQP4 in lesions, while remaining preserved in myelinated fibers (117). This finding supports the view that demyelination is secondary to astrocytic damage. However, some inflammatory lesions in the spinal cord also show non-lytic alterations in GFAP-positive reactive astrocytes, implicating an early AQP4-targeted attack. This observation supports AQP4 loss prior to astrocyte loss (112) but is contrary to the above view that AQP4 loss in NMO lesions is secondary to astrocyte loss. Hence, the exact pathogenetic mechanism of AQP4 in NMOSD requires further investigation. It is important to identify therapeutic targets to prevent or relieve the symptoms of NMOSD.

### The Interaction Between NMOSD and Pregnancy

NMOSD develops predominantly at childbearing age, and the interaction between NMOSD and pregnancy deserves to be mentioned (118). Unlike MS, NMOSD is exacerbated acutely during pregnancy (119). However, large-scale studies have shown that the influence of pregnancy in NMOSD is significantly higher during the first 3 months, with no reduction of the annualized relapse rate in the third trimester (118, 120, 121).

**Figure 2:**
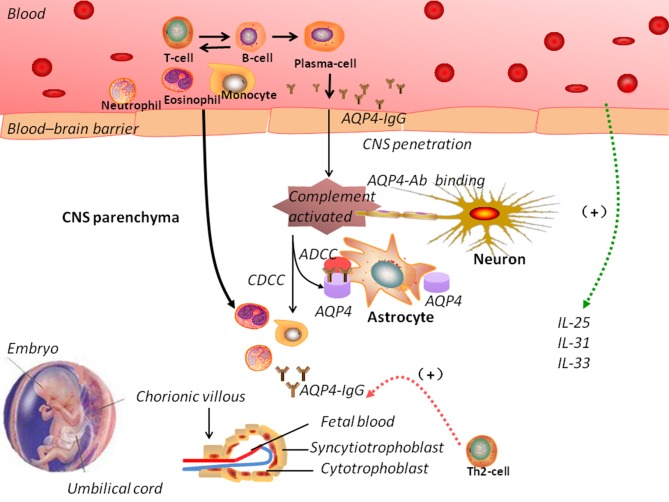
The pathogenic mechanisms of neuromyelitis optica spectrum disorders (NMOSD). In NMOSD, the peripheral circulation contains autoantibodies against aquaporin-4 (AQP4-IgG). Disruption of the blood–brain barrier (BBB) allows the entry of AQP4-IgG into the central nervous system. Increased BBB permeability induced by complement activation could trigger the infiltration of eosinophils and neutrophils. Antibody-dependent astrocyte damage involving CDCC and ADCC mechanisms causes oligodendrocyte injury, demyelination, and neuronal loss. During pregnancy, normal trophoblast cells have specific embryonal antigens that account for the “foreignness” of an allograft; a shift toward Th2-mediated immunity could lead to increased antigen stimulation and production of NMO-IgG, which could explain the pathogenesis of NMOSD. However, the interaction between NMOSD and pregnancy remains elusive. ADCC: antibody-dependent cellular cytotoxicity; CDCC: complement-dependent cellular cytotoxicity.

Pregnancy can induce immunological and hormonal changes, including a major shift from Th1-mediated to Th2-mediated immunity (122). In NMOSD, the number of Th1 cells is reduced and anti-inflammatory cytokines are produced, resulting in a shift toward Th2-mediated immunity, which affects the pathogenesis of the disease. A shift toward Th2 in pregnancy can lead to increased antigen stimulation and production of NMO-IgG (123). Figure 2 shows the possible mechanisms of NMOSD pathogenesis during pregnancy. However, Quick and Cipolla (124) showed an up-regulation of the AQP4 in the CNS during pregnancy in mice. Saadoun et al. (125) reported a miscarriage rate of 13% in patients NMOSD and discovered that NMO-IgG triggers placentitis, causing fetal death in mice.

## Conclusion

Pregnancy is associated with changes in the immune system, which can affect the outcome of various diseases, particularly those of the CNS. Different types of demyelinating diseases have their own characteristics, and the complex immune changes that occur during pregnancy can have different effects on the progression and prognosis of the disease. This review confirmed a lower relapse rate of MS during pregnancy, with an increase in the relapse rate during the postpartum period. Pregnancy can accentuate the symptoms of ADEM, and can promote acute exacerbation or recurrence of NMOSD after delivery. However, our understanding of the contribution of pregnancy to the immune-pathologic mechanisms remains limited. The relationship between demyelinating diseases of the CNS and pregnancy in relation to contribute to those processes.

## Author Contributions

WL conceived and designed the study. KQ wrote the paper. QH, XC, HL, and SD reviewed and edited the manuscript. All authors read and approved the manuscript.

### Conflict of Interest Statement

The authors declare that the research was conducted in the absence of any commercial or financial relationships that could be construed as a potential conflict of interest.
